# Rapid development and deployment of high‐volume vaccines for pandemic response

**DOI:** 10.1002/amp2.10060

**Published:** 2020-06-29

**Authors:** Zoltán Kis, Cleo Kontoravdi, Antu K. Dey, Robin Shattock, Nilay Shah

**Affiliations:** ^1^ Centre for Process Systems Engineering, Department of Chemical Engineering, Faculty of Engineering Imperial College London London UK; ^2^ International AIDS Vaccine Initiative (IAVI) New York New York USA; ^3^ Department of Infectious Disease, Faculty of Medicine Imperial College London UK

**Keywords:** bioprocess modeling, distributed manufacturing, pandemic‐response vaccine manufacturing, Quality by Design, RNA vaccines, techno‐economic modeling, vaccine platform technology

## Abstract

Overcoming pandemics, such as the current Covid‐19 outbreak, requires the manufacture of several billion doses of vaccines within months. This is an extremely challenging task given the constraints in small‐scale manufacturing for clinical trials, clinical testing timelines involving multiple phases and large‐scale drug substance and drug product manufacturing. To tackle these challenges, regulatory processes are fast‐tracked, and rapid‐response manufacturing platform technologies are used. Here, we evaluate the current progress, challenges ahead and potential solutions for providing vaccines for pandemic response at an unprecedented scale and rate. Emerging rapid‐response vaccine platform technologies, especially RNA platforms, offer a high productivity estimated at over 1 billion doses per year with a small manufacturing footprint and low capital cost facilities. The self‐amplifying RNA (saRNA) drug product cost is estimated at below 1 USD/dose. These manufacturing processes and facilities can be decentralized to facilitate production, distribution, but also raw material supply. The RNA platform technology can be complemented by an a priori Quality by Design analysis aided by computational modeling in order to assure product quality and further speed up the regulatory approval processes when these platforms are used for epidemic or pandemic response in the future.

## 1 INTRODUCTION

Vaccines are considered the most effective form of healthcare intervention^[^ ^1^, ^2^ ^]^ and offer the promise of overcoming the current Covid‐19 pandemic caused by the SARS‐CoV‐2 virus. One of the most promising technologies for the development of an effective SARS‐CoV‐2 vaccine are considered to be RNA and viral vector‐based platforms.^[^ ^3^, ^4^, ^5^ ^]^ The RNA vaccine platform uses the natural cellular protein expression pathway, based on the central dogma of molecular biology, in which genetic information encoded in DNA is transcribed into messenger RNA (mRNA) and translated into protein. This way, RNA vaccinology works by outsourcing the production of the vaccine protein antigen to the cells of the human body, based on the information in the RNA sequence.^[^ ^6^, ^7^, ^8^, ^9^ ^]^ For this, the RNA vaccine is commonly injected into the muscle using predominantly a liposome‐based formulation, known as lipid nanoparticle, or a polycation‐based formulation.^[^ ^7^, ^9^ ^]^ Once inside the cells, the ribosomes produce the protein encoded by the RNA sequence, which for the Covid‐19 vaccine is the spike protein on the SARS‐CoV‐2 virus surface. The produced protein antigen (expressed as spike protein trimer) then induces the immune response likely required to gain immunity against the virus. There are two main types of RNA vaccines, mRNA^[^ ^7^ ^]^ and self‐amplifying RNA (saRNA) vaccines.^[^ ^8^, ^10^ ^]^ As their name implies, saRNA vaccines, replicate inside cells by encoding a viral RNA replication machinery in the saRNA strand and expressing this inside the human cells.^[^ ^8^, ^10^ ^]^ This way, lower amounts of RNA are required per vaccine dose, potentially providing substantial cost benefits and higher productivity, in terms of doses per liter of bioreaction, compared to non‐replicating mRNA vaccines.^[^ ^10^, ^11^ ^]^ On the other hand, mRNA vaccines are clinically more developed and widely tested compared to saRNA vaccines. Viral vector‐based vaccines, such as Adenovirus vector vaccines, also utilize the cells of the human body to synthesize the target antigen, however they deliver a DNA payload,^[^ ^12^ ^]^ which is first transcribed into an mRNA and then translated into the spike protein in the case of the Covid‐19 vaccine. With clinical trials of RNA vaccines currently ongoing, herein we conduct a techno‐economic analysis of RNA vaccine manufacturing and present the advantages of this platform with respect to development speed, manufacturing footprint and vaccine cost.

## 2 ASSESSMENT OF THE RNA VACCINE PLATFORM

### 2.1 Development timeline

**FIGURE 1:**
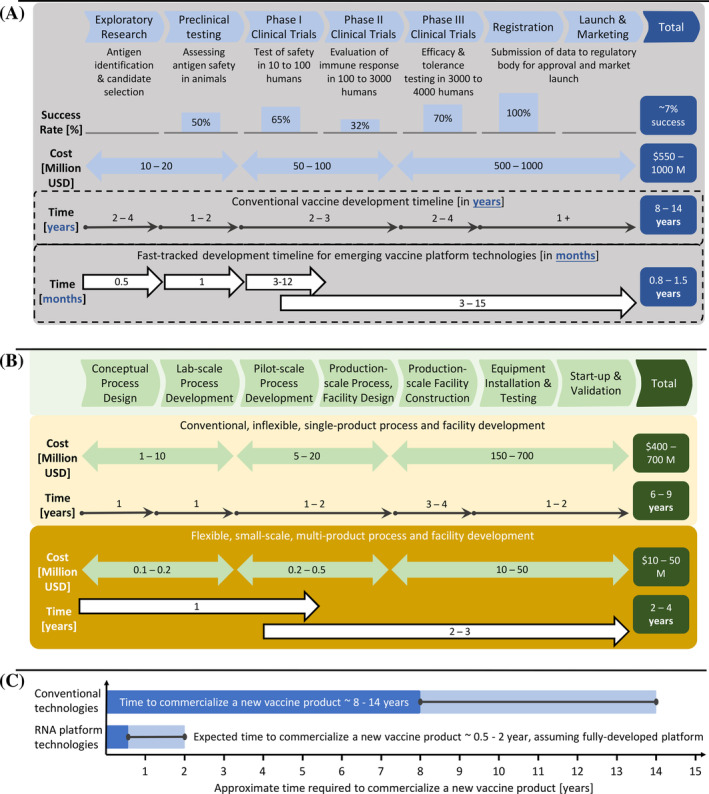
Overview of vaccine testing and manufacturing development timelines. A, Pre‐clinical development and clinical testing timelines, costs and success/failure rates for conventional vaccines and for vaccines produced using emerging platform technologies (eg, RNA vaccines).^[^ ^13^, ^14^, ^15^, ^16^, ^17^, ^18^, ^19^, ^20^, ^21^, ^22^ ^]^ Once the platform is developed and used to produce a licensed product, the costs and failure rates for developing further products using the same platform technology would drop substantially. The highest costs and longest development timelines are encountered in phase III clinical trials and the highest failure rates tend to occur in phase II clinical trials. B, Process development and facility construction timelines and cost estimates for conventional and emerging platform technologies with drug substance annual production capacities of tens to hundreds of million doses.^[^ ^14^, ^19^, ^40^, ^41^, ^42^, ^59^ ^]^ Process development and facility design is usually initiated during pre‐clinical and clinical testing and investments are usually made as failure risks reduce during clinical development. C, Comparison of overall vaccine production rates for conventional and new platform technologies, considering the development and testing phases presented in parts A and B above. Once fully developed and validated, the new vaccine platform technologies will produce vaccines within weeks to months after antigen identification, which is at least 10‐fold faster than conventional technologies

Conventional vaccine development takes on average 8 to 14 years and costs 0.55 to 1 billion USD,^[^ ^13^, ^14^, ^15^, ^16^, ^17^, ^18^, ^19^, ^20^, ^21^, ^22^ ^]^ as illustrated in Figure 1. Fast‐tracked regulatory processes implemented for emergency response to pandemics can cut the duration of pre‐clinical and clinical development to 0.8 to 1.5 years if patient recruitment and testing can be carried out rapidly.^[^ ^21^, ^23^ ^]^ Emerging platform technologies, such as the RNA platform, promote pre‐clinical development at unprecedented speeds.^[^ ^24^, ^25^ ^]^ For example, the Shattock group at Imperial College London generated a prototype saRNA vaccine candidate 2 weeks after the selection of the genetic sequence of the spike protein from the SARS‐CoV‐2 virus,^[^ ^3^, ^26^ ^]^ and US company Moderna, Inc. went from genetic sequence information of the clinical material manufacturing to human testing in only 42 days.^[^ ^22^ ^]^ These speeds to clinical material production provide huge advantage to clinical investigation of multiple vaccine candidates in face of a pandemic. During clinical development, the highest costs and longest durations are encountered in phase III clinical trials and the highest failure rates tend to occur in phase II clinical trials where the efficacy of the vaccines is assessed,^[^ ^14^, ^20^, ^27^, ^28^ ^]^ cf. Figure 1A.

Once the cGMP platform production process for RNA vaccines is developed, RNA vaccines can be produced substantially faster compared to conventional expression systems. For example, in the case of inactivated or live‐attenuated viral (such as the PiCoVacc SARS‐CoV‐2 virus vaccine candidate being produced in Vero Cells), or recombinant protein vaccine candidate production, product‐specific manufacturing processes have to be developed and ideally optimized, validated and approved for cGMP production, which can take a substantial amount of time. Additionally, the agility of the RNA platform, which is agnostic to the disease target, means that multiple iterations and vaccine variants can be rapidly produced and tested without the need for process modification or re‐validation.

The high productivity of the RNA platform as expressed per unit volume of process and per unit time is also considerably higher than the aforementioned conventional expression systems. This makes the production of higher volumes required for later phase trials and for large scale production substantially easier. The time and resource gains to be made with the RNA platform are expected to become more apparent in the future when this platform is fully developed and RNA vaccines gain regulatory approval.

### 2.2 Manufacturing process and footprint

Following successful clinical trials and demonstration of efficacy, the next challenge becomes the manufacturing of the vaccine at the quality standards, scale and rate required for meeting global demand. This is particularly cumbersome in the case of pandemic‐response manufacturing, when several billion doses of vaccines need to be manufactured within months under current Good Manufacturing Practices (cGMP), and ideally at low enough cost to allow affordability and mass immunization globally. Small‐scale cGMP compliant RNA vaccine production processes have been already developed and GMP grade RNA vaccine candidates have already been produced for clinical trials.^[^ ^29^, ^30^, ^31^, ^32^, ^33^ ^]^ In the light of the current COVID‐19 pandemic, several companies and consortia are scaling up RNA vaccine production to the billion dose annual production scale.^[^ ^34^, ^35^, ^36^, ^37^, ^38^, ^39^ ^]^

For rapid response manufacturing, optimal utilization of existing facilities is crucial, however if the demand cannot be met, the construction of additional facilities will be urgently required. Crucially, manufacturing a new vaccine of conventional format (eg, inactivated viral vaccines) against a new disease, such as Covid‐19, by re‐deploying existing large‐scale facilities would also adversely affect the supply of other medicines. With the construction of such conventional facilities requiring around several years and hundreds of million US dollars^[^ ^14^, ^19^, ^40^, ^41^, ^42^, ^43^, ^44^ ^]^ (Figure 1B **)**, certain emerging platform technologies present considerable advantages for rapid‐response manufacturing.^[^ ^24^ ^]^

**FIGURE 2:**
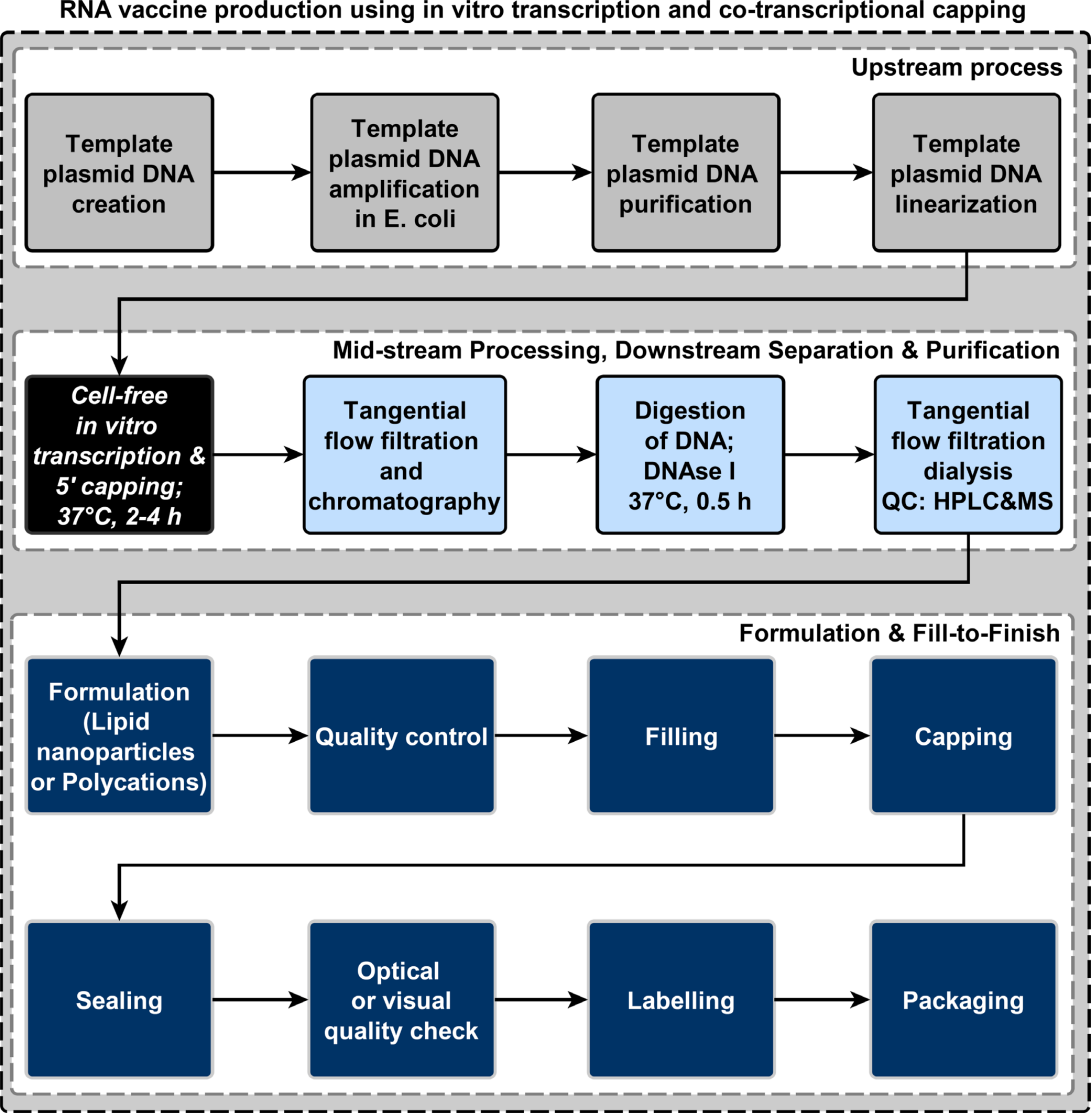
Process flow diagram for saRNA vaccine production based on the in vitro transcription enzymatic reaction. In the upstream process the DNA template is generated, amplified, purified and linearized. In the mid‐stream process the RNA is synthesized following the in vitro transcription reaction using the T7 RNA polymerase enzyme, and 5′ capping of the RNA is achieved co‐transcriptionally using 5′ cap analogues (needed to ensure antigen expression). For downstream purification TFF can be used also in combination with chromatography methods, such as hydroxyapatite chromatography and core bead flow‐through chromatography. In the first TFF step the saRNA and linearized DNA template are retained by the filter and smaller molecular size components, including the T7 RNA polymerase enzyme, flow through the filter. Next, the linearized DNA template is digested using nucleases and then the DNA nucleotides can be separated from the RNA using another TFF step. The obtained drug substance is then formulated predominantly in lipid nanoparticles, however polycationic formulations are also developed and evaluated. Next, the formulated saRNA undergoes quality control and is filled into vials or containers for pandemic‐scale mass vaccination. The vials are then capped, sealed, inspected using automated image processing, labeled and packaged into secondary and tertiary packaging. The entire production process is independent of the RNA sequence, therefore in principle vaccines against virtually any disease can be produced using the same production process^[^ ^24^, ^46^, ^48^, ^49^, ^60^ ^]^

We have built a production process model for RNA vaccine manufacturing in SuperPro Designer (Intelligen, Inc.) based on state‐of‐the‐art processes, implemented in industry for RNA synthesis utilizing DNA‐templated enzymatic RNA synthesis via the in vitro transcription reaction catalysed by the T7 RNA polymerase enzyme,^[^ ^7^, ^45^, ^46^ ^]^ cf. the process diagram in Figure 2. The saRNA vaccine drug substance encoding for the spike protein from the SARS‐CoV‐2 has a size of around 10 k bases (kb) and a molecular mass of around 5 Mega Dalton (MDa), which is much larger when compared to the mRNA vaccine drug substance of the same spike‐encoding protein that has a size of around 3 to 4 kb, corresponding to 1.5 to 2 MDa.^[^ ^5^, ^47^ ^]^ The molecular masses of saRNA and mRNA are an order of magnitude larger than the T7 RNA polymerase enzyme and therefore size‐based separation of the T7 RNA polymerase from the RNA seems feasible. After RNA synthesis, downstream purification can be achieved via a series of tangential flow filtration (TFF) steps, and purities of 90% to 99.9% and yields of 90% to 95% have been reported.^[^ ^48^ ^]^ TFF can also be complemented by chromatographic purification techniques, such as hydroxyapatite chromatography, oligo(dT) chromatography, ion exchange chromatography and core bead flow‐through chromatography (eg, Capto Core 700 beads from Cytiva, Danaher Corporation, formerly GE Healthcare Life Sciences).^[^ ^48^, ^49^ ^]^ The produced RNA drug substance is then formulated into lipid nanoparticles^[^ ^3^, ^9^, ^50^ ^]^ to complete the production of the LNP‐encapsulated/formulated RNA. The overall LNP‐encapsulation process and formulation is being independent of the RNA sequence and, thus the targeted disease vaccine drug product. Once the formulated RNA is ready, it enters the fill‐to‐finish process where in the required dose (plus overage) are filled into glass vials to produce the final vaccine drug product.

Based on our techno‐economic assessment, the RNA vaccine production process can be two to three orders of magnitude smaller than conventional vaccine production processes in terms of facility scale, and can be constructed in less than half the time with 1/20 to 1/35 of the upfront capital investment, as shown in Figure 1B. It therefore presents a strong advantage of requiring small‐scale, high‐capacity facilities, which can be constructed more rapidly and could make wide use of single‐use disposable equipment. Due to its small scale, the RNA vaccine drug substance production process could be placed in a small part of an existing conventional vaccine facility, for example in a room, and still produce more doses worth of drug substance than the entire original conventional vaccine production facility. To rapidly establish such an RNA vaccine drug substance production line, off‐the‐shelf single‐use equipment can be used to build the entire process. Once such a process is established and validated based on readily available single‐use equipment, the technology can be transferred to other facilities for scaling out purposes, thereby reducing process and quality control design and development timelines and streamlining validation and start‐up activities.

Moreover, the RNA vaccine platform technology offers the flexibility of producing a very large range of vaccine products using the same production process, quality control system and facility, rapidly and at high capacity. Therefore, the production of new vaccines can be achieved around 10× faster compared to conventional vaccine production technologies, as shown in Figure 1C. In such a scenario, the cost of an RNA vaccine drug substance manufacturing facility, besides scale also depends on the grade of the clean rooms or required containment level. If the RNA production can take place in a closed system,^[^ ^48^ ^]^ then lower grade facilities and rooms can even be used, which would cost substantially less to construct, operate and maintain compared to high grade clean room containing facilities, of course, following the appropriate regulatory and compliance guidelines.^[^ ^49^ ^]^

### 2.3 Process performance and costs

Based on our process‐cost modeling in SuperPro Designer (the model, relevant assumptions and simulation results report are available on GitHub at https://github.com/ZKis-ZK/RNA-vaccine-drug-substance-production-techno-economic-modelling), the saRNA platform can produce over 1 billion vaccine doses worth of drug substance per year at a small process scale corresponding to 5 L bioreactor working volume in a correspondingly small facility footprint that would cost around 20 million USD to construct, equip, validate and start up. The annual operating costs are estimated to be over 100 million USD due to the high cost of raw materials involved in RNA synthesis and LNP production. This 100 million USD/year estimate includes material and consumable costs, labor costs, facility‐dependent costs, quality control and quality assurance costs, and waste disposal costs. Out of these, the 5′ cap analogue raw material is the major cost component, accounting for over 50% of the total operating costs. The highest‐efficiency 5′ cap analogues are CleanCap AG and CleanCapAU (TriLink Biotechnologies, Inc.) for mRNA and saRNA vaccines, respectively.^[^ ^51^ ^]^ The 5′ cap structure is crucial for avoiding degradation of the RNA by the innate immune mechanisms, which associate non‐capped RNA with foreign (eg, viral) material and ensures protein antigen expression from the RNA polymer.^[^ ^52^ ^]^ The production of 5′ cap analogues is currently being scaled up to ensure availability for billion‐dose scale RNA vaccine production.^[^ ^53^ ^]^ We therefore do not envisage the availability of 5′ cap analogues to be a bottleneck, particularly for saRNA production, although raw material shortages cannot be ruled out during pandemic‐response mass vaccine manufacture.

The amount of RNA drug substance per dose is estimated to be in the range of 0.1 to 10 μg/dose^[^ ^3^, ^10^, ^11^ ^]^ and 25 to 250 μg/dose^[^ ^3^, ^54^ ^]^ for saRNA and mRNA vaccines, respectively. The actual amount will be determined during clinical trials. Given this difference and range in the amount of RNA drug substance per dose, the price per dose, production amounts and production rates will vary accordingly. This is in line with the original intended purpose of these vaccines: mRNA vaccines were originally developed as anti‐cancer vaccines without prioritizing ultra‐low cost per dose.^[^ ^5^, ^6^, ^10^ ^]^ On the other hand, saRNA vaccines have been developed for infectious diseases and rapid pandemic response, considering the purchasing power of developing countries and thus aiming to minimize cost per dose.^[^ ^5^, ^6^, ^10^ ^]^ Based on our cost modeling, a cost per dose of below 1 USD/dose, including options for fill‐to‐finish into multidose vials, appears achievable for saRNA vaccines. This cost could be well above 1 USD/dose for mRNA vaccines. The drug substance cost per dose and productivity, in terms of doses worth of drug substance produced per unit time, has a linear dependence on the drug substance amount per dose. This way, the drug substance cost per dose can decrease and productivity can increase by two orders of magnitude in case of moving from 10 to 0.1 μg/dose for saRNA vaccines. As listed in Table 1, besides the RNA amount per dose, the cost per dose and production process performance for both mRNA and saRNA vaccines will depend on the process scale, production titre, cost of the 5′ cap analogue or the 5′ capping approach used (co‐transcriptional vs enzymatic post‐transcriptional),^[^ ^7^ ^]^ efficiency and cost of downstream purification methods, the facility‐related costs and the possibility of recycling high value materials for producing a subsequent batch of the same product. The time required to produce a batch of RNA drug substance is around 11 hours, if production batches are scheduled such that the end of a batch overlaps with the beginning of the subsequent batch in order to increase the utilization of the production line. Continuous RNA synthesis production processes whereby the RNA product is continuously exiting the bioreactor and the high value raw materials are kept in the bioreactor also offer a substantial material cost reduction potential.^[^ ^55^ ^]^ Besides the manufacturing costs, the final sale price of the vaccine is also expected to include R&D costs, costs of clinical trials, marketing and supply chain costs and a profit margin.

**TABLE 1:** Parameters influencing RNA vaccine production performance and cost

Parameter	Range	Unit	Influencing and determining factors	Reference
RNA amount per dose	0.1‐10 for saRNA 25‐250 for mRNA	μg/dose	Clinical trials	3, 10, 11, 54 α
Process scale	0.5‐50	L	Demand, scale‐up optimization	46, 49, 55, 60
Production titres	1.5‐7	g/L	Reaction optimization, process development	46, 55
5′ Cap analogue cost	2500‐10 000	USD/g	Scale and supplier purchase price	53
Downstream purification losses	20‐50	%	Type of unit operations, process development	48, 49, 60 β
Raw material recycling^a^	0‐8	Fold	Stability of the materials, regulatory approval	α
Capital investment costs	10‐40	Million USD	Production scale, grade and containment level of the facility	β
Vial or container cost	0.1‐0.6	USD/dose	Number of doses per container or vial	56, 61

*Note*: α—Assumed by the authors; β—calculated using SuperPro Designer V10 (Intelligen, Inc.).

^a^ Recycling or re‐use of the materials for producing multiple batches of the same product. For this, high cost raw material (eg, the 5′ cap analogue and enzymes) can be separated from the RNA product using TFF and fed back into the RNA synthesis bioreactor.

## 3 CHALLENGES AHEAD AND POTENTIAL SOLUTIONS

The biggest challenge for saRNA and mRNA vaccines currently is to demonstrate efficacy against target disease in clinical trials, especially the Phase II/III efficacy trials, where the highest proportion of vaccines tend to fail. However, the success rate during clinical trials can be increased and the clinical development timelines can in principle be reduced, because the generic feature of the RNA platform allows for the rapid production and development of many vaccine variants, allowing for rapid iterations during clinical development. Additionally, there are numerous intellectual property challenges to be solved, including the ones related to LNP‐based formulation. However, it seems that there are no technical or scalability bottleneck related to current formulation processes when considering high‐volume pandemic‐response production. On the other hand, formulation raw material availability can become a bottleneck during high‐volume pandemic‐response vaccine production, however, their production is also being scaled up to meet global demand. Pandemic‐induced transportation disruptions can limit the availability of raw materials, impacting specialty materials more substantially due to the lower number of suppliers. To mitigate this, distributed or outsourced manufacturing of raw materials across several continents could offer a solution. The stability of the vaccine can also influence its global distribution and availability. This way, vaccines which are stable and require distribution at −70°C, would limit distribution in low and middle income countries due to the lack of the appropriate cold chain infrastructure, would limit the use of multidose vials, and would increase the cost per dose due to cold chain distribution costs and because of the single dose vial (or low dose number) format. To overcome this issue, vaccine developers are evaluating formulations with higher thermostability or lyophilized formulations.

Finally, there is a need for additional manufacturing capacity for pandemic‐response production. However, the RNA vaccine production processes are extremely productive, especially for saRNA vaccines due to lower expected amount per dose. With the annual production of saRNA drug substance for over 1 billion doses using a 5 L bioreactor working volume, the bottleneck is expected to be the fill‐to‐finish process that may not be able to fill billions of vials with formulated RNA, at room temperature, particularly when the formulated RNA may not be stable at that condition for a prolonged period of time. To address this, 200 dose bags are being evaluated by CEPI, which can be filled at a rate of 3 million vaccine doses during an 8 to 10 hours manufacturing work shift.^[^ ^56^, ^57^ ^]^ This will potentially add complexity to the final vaccine administration step in clinic where the pharmacists and medical professionals will need familiarity with the new requirements of this technology.

**FIGURE 3:**
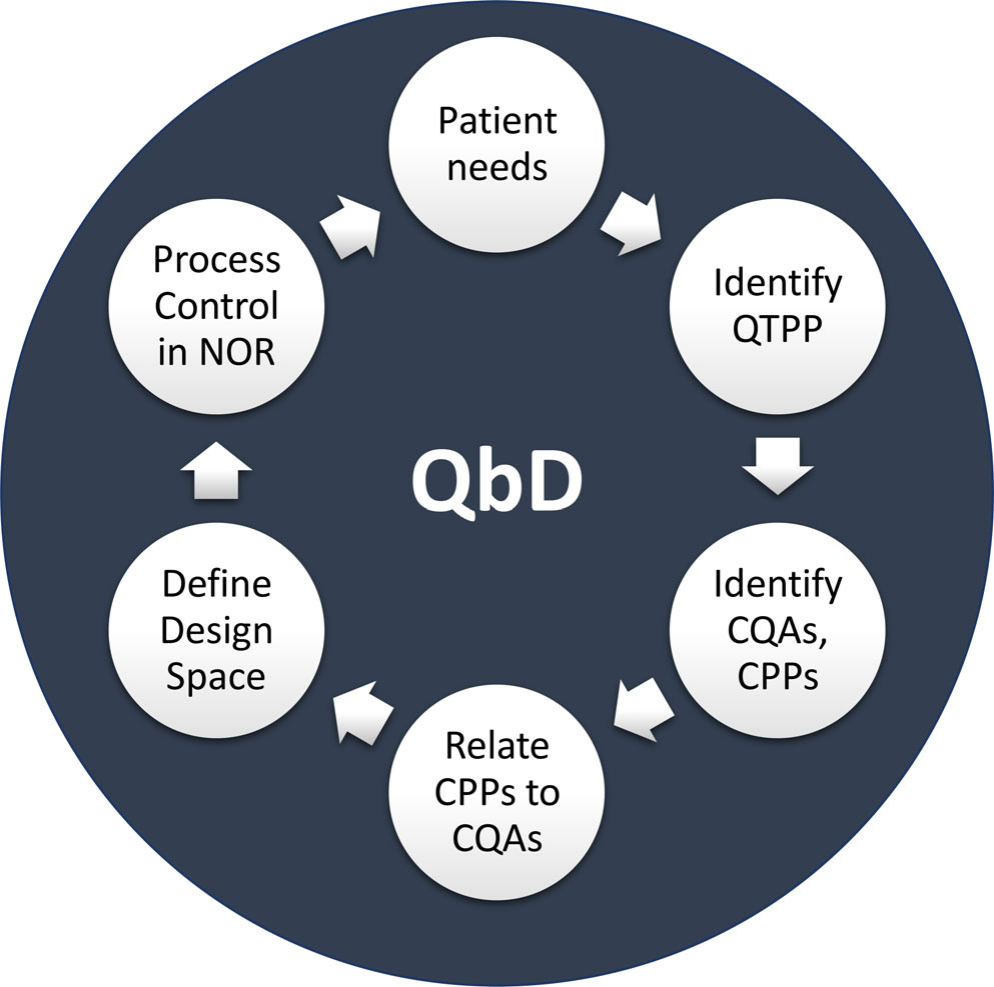
Quality‐by‐design (QbD) framework. The QbD development cycle begins with identifying the patient needs and based on these the Quality Target Product Profile (QTPP) is defined. From the QTPP, the critical quality attributes (CQAs) of the product and their ranges are determined using a risk assessment scoring, based on clinical and non‐clinical data, for both safety and efficacy. Next, based on the CQAs and on understanding the of production process, the critical process parameter (CPP) ranges are defined. Mathematical relations between CPPs and CQAs are established, obtaining this way a mathematical model of the vaccine production process. Using this model, the ranges of CPPs which yield the desired CQAs are determined. Based on these CPP ranges, the design space is determined and therein a sub‐space called the normal operating range (NOR) is defined. The NOR offers the flexibility of modifying operating parameters in the GMP production process, thus allowing optimization to account for inherent biological heterogeneity, instead of “freezing” the GMP process. The QbD bioprocess model can be adapted for advanced process control, using model predictive control and real‐time measurement data from the production process. Such a “digital twin” model can predict CQA values in the following time window (eg, next 5 minutes) and if CQAs are predicted violate the specified ranges, the model can recommend corrective measures, that is, control actions, to prevent CQAs going out of the specified ranges, fixing mistakes before these occur. Thus, computational modeling tools can be integrated with experimental development and QbD follows an iterative development cycle to ensure continuous improvement through the product‐process life cycle^[^ ^58^ ^]^

Once the saRNA and mRNA platforms are fully developed, stability issues resolved, manufacturing and operational processes addressed and pandemic‐ready, the regulatory processes could, in principle, be accelerated to rapidly respond to future epidemics and pandemics by applying a Quality by Design (QbD) framework aided by computational modeling, cf. Figure 3. QbD is a systematic approach to pharmaceutical product development that begins with pre‐defined objectives and integrates product and process understanding, identification of critical quality attributes (CQAs) and critical process parameters (CPPs), product quality risk management and development of robust control strategy to ensure the quality of the product.^[^ ^58^ ^]^ For accelerated development, this framework would incorporate bioprocess modeling, soft sensing and advanced process control in order to consistently ensure the quality of the manufactured drug substance and drug product. The RNA platform technologies can be supplemented with the above mentioned QbD framework, which could be applied universally and a priori to assure the quality and speed during development of any RNA vaccine production process, independently of the RNA sequence and disease target.

To develop such a QbD framework, the iterative cycle described in Figure 3 can be employed. For this the Quality Target Product Profile (QTPP) can be determined based on the optimal profile of the vaccine to meet patient needs and provide highest level of protection. Then the critical quality attributes (CQAs) of the product and their ranges can be determined based on the QTPP, by using a risk assessment scoring. Next, critical process parameter (CPP) ranges can be defined based on the CQAs and on the understanding of the production process. Next mathematical relations between CPPs and CQAs can be established, thereby obtaining a mathematical model of the vaccine production process. This model can be data‐driven, statistical, mechanistic or hybrid and, in all cases, it can be further calibrated and then validated with experimental data. Mechanistic models or mechanistic components of a hybrid model usually tend to provide more predictive power than data‐driven mathematical descriptions. By running the validated mathematical model, the ranges of CPPs which yield the desired CQAs can be determined and, based on these, the design space can be established. Within the design space, a sub‐space, called the normal operating range (NOR), can be defined that offers the flexibility of modifying and optimizing operating parameters in the GMP production process, rather than “freezing” the cGMP process. The model can be simplified and adapted for advanced automation, based on model predictive control, which uses real‐time data from the production processes. By coupling such a modeling‐aided QbD framework to the saRNA or mRNA platforms, the quality aspects of RNA‐product can be harmonized, and regulatory processes could, in principle, be accelerated. In addition, this modeling‐aided QbD framework can also ensure better management of product quality risks during scale‐up and subsequent manufacturing.

During a pandemic, manufacturing and supply chain challenges can occur due to lockdowns, closures of upstream manufacturing facilities (eg, raw material, consumable and single‐use equipment manufacturing), reduction of labor force due to health issues caused by the outbreak, travel and transportation restrictions and due to contamination risks of input materials. These threats can cause more severe sourcing disruptions in case of centralized manufacturing and supply chains, due to the reliance on a small number of key manufacturing facilities and supply chain routes. This can be partially addressed by maintaining adequate stock levels and more appropriately addressed by the implementation of distributed, that is, decentralized, manufacturing and supply chains. This way, the number of facilities and supply routes could increase, therefore reducing the risk of lack of raw materials and consumables at a single location (although we note that the decentralized system creates more complex inbound material supply chains), and ultimately increasing the probability of the sustained vaccine supply.

## 4 CONCLUSIONS

To address pandemics, such as the Covid‐19, several billion doses of vaccines are needed within months. Emerging outbreak‐response platform technologies, especially the RNA platform, appear capable of addressing this extremely challenging task, due to their high productivity at low manufacturing footprint and its ability to” release” millions of doses through rapid quality control testing. Specifically, a facility with a single 5 L bioreactor working volume can be sufficient to produce an estimated 1 billion vaccine doses per year at a drug product cost of below 1 USD/dose. This further increases the possibility of distributed manufacturing and thus contributing to vaccine supply sustainability. Given that RNA vaccine production processes are two to three orders of magnitude smaller than conventional vaccine production processes, they can be built in less than half the time with 1/20 to 1/35 of the upfront capital costs compared to conventional vaccine production processes. Inclusion of single‐use technologies in RNA vaccine manufacturing can further accelerate this timeline. Once the RNA production platform is established, the overall clinical development process could, in principle, be further accelerated by the utilization of a computational model‐aided QbD platform, which would complement the platform production, independent of the RNA sequence. This would enable the development of a platform process agnostic to the infectious disease target, which can be rapidly deployed to both develop candidate vaccines for testing and large‐scale manufacture. Despite the numerous production and affordability advantages, the RNA platform still remains unproven with no commercial vaccine developed using this “disruptive” technology. As multiple organizations exploit this technology to develop vaccines against SARS‐CoV‐2, the benefits and practical limitations of this technology will be tested, providing lessons for further iteration and improvements.
